# Smart Strategies to Overcome Drug Delivery Challenges in the Musculoskeletal System

**DOI:** 10.3390/ph16070967

**Published:** 2023-07-06

**Authors:** Brandon Vorrius, Zhen Qiao, Jonathan Ge, Qian Chen

**Affiliations:** Laboratory of Molecular Biology and Nanomedicine, Department of Orthopaedics, Alpert Medical School of Brown University/Rhode Island Hospital, Providence, RI 02903, USA; brandon_vorrius@alumni.brown.edu (B.V.); qinchuanissb@gmail.com (Z.Q.); jonathan_ge@brown.edu (J.G.)

**Keywords:** musculoskeletal system, drug delivery, tissue barriers, cartilage, muscle, bone

## Abstract

The musculoskeletal system (MSKS) is composed of specialized connective tissues including bone, muscle, cartilage, tendon, ligament, and their subtypes. The primary function of the MSKS is to provide protection, structure, mobility, and mechanical properties to the body. In the process of fulfilling these functions, the MSKS is subject to wear and tear during aging and after injury and requires subsequent repair. MSKS diseases are a growing burden due to the increasing population age. The World Health Organization estimates that 1.71 billon people suffer from MSKS diseases worldwide. MSKS diseases usually involve various dysfunctions in bones, muscles, and joints, which often result in pain, disability, and a decrease in quality of life. The most common MSKS diseases are osteoporosis (loss of bone), osteoarthritis (loss of cartilage), and sarcopenia (loss of skeletal muscle). Because of the disease burden and the need for treatment, regenerative drug therapies for MSKS disorders are increasingly in demand. However, the difficulty of effective drug delivery in the MSKS has become a bottleneck for developing MSKS therapeutics. The abundance of extracellular matrix and its small pore size in the MSKS present a formidable barrier to drug delivery. Differences of vascularity among various MSKS tissues pose complications for drug delivery. Novel strategies are necessary to achieve successful drug delivery in different tissues composing the MSKS. Those considerations include the route of administration, mechanics of surrounding fluids, and biomolecular interactions, such as the size and charge of the particles and targeting motifs. This review focuses on recent advances in challenges to deliver drugs to each tissue of the MSKS, current strategies of drug delivery, and future ideas of how to overcome drug delivery challenges in the MSKS.

## 1. Introduction

### 1.1. MSKS Tissue Matrix Forms Barriers of Drug Delivery

The MSKS is an integrated system of various specialized connective tissues that achieve specific structural functions. For example, the knee joint consists of (1) articular cartilage that covers the joint bone structure; (2) the meniscus, which serves as a cushion for the opposing articular surfaces; (3) subchondral bone beneath the articular cartilage; (4) the ligament that connects bone to bone and the tendon that connects bone to muscle; and (5) skeletal muscle that surrounds the bone. Each of these MSKS tissues have different, independent structures and functions, which coordinate to create the structural integrity and mobility of the knee joint. The extracellular matrix (ECM) is highly enriched in MSKS tissues due to their mechanical functions. The ECM also serves as a barrier for the diffusion of molecules such as drugs to reach cells. Just like each tissue’s structure and function, the ECM organizational characteristics such as pore size and surface charge vary among the MSKS. Thus, the therapeutic delivery strategy needs to vary accordingly.

### 1.2. Vascularity

In addition to ECM, there are differences in vascularity among MSKS tissues. Due to the high demand of structural adaptation and regeneration, there is high vascularity in bone and muscle. On the other hand, cartilage, tendon, and ligament have low vascularity due to their structural properties that prioritize low tissue turnover and structural stability. Another consideration is that bone and muscle diseases such as osteoporosis and sarcopenia are usually systematic and affect the whole body. In these cases, systemic drug delivery is preferred because it reaches different sites of the body rather than just a single bone or muscle. High vascularity in bone and muscle also provides advantages for drugs to reach these tissues through blood vessel transport. On the other hand, degeneration of cartilage, tendon, and ligament is often local, affecting one or more joints. The lack of vascularity in these tissues also renders systemic drug delivery ineffective. Intra-articular injection is usually employed for local delivery of therapeutics for the treatment of these tissues. The considerations for systemic vs. local administration are described as follows.

## 2. Route of Administration

**Figure 1:**
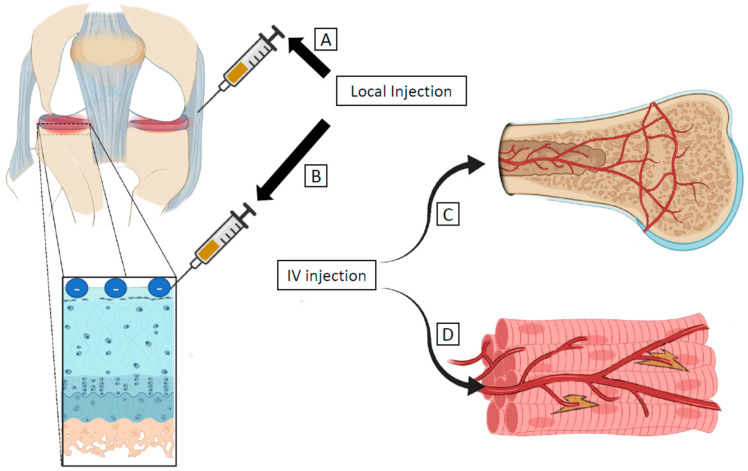
Administration routes targeting avascular and vascular tissues in the MSKS. Local delivery such as intra-articular injection is often used to target avascular tissues including tendon and ligament (**A**) and cartilage (**B**). Systemic delivery such as intravenous (IV) injection is often used for drug delivery to vascular tissues such as bone (**C**) and muscle (**D**).

In this section, we describe the advantages of local versus systemic deliveries (Figure 1). The disease pathophysiology, target tissue or cell type, and therapeutic composition influence the chosen route of delivery. Based on these features, the MSKS actively compartmentalizes particles into different tissues through its tissue barrier function. Certain aspects of the MSKS tissue barrier act like physiologic barriers in the brain, gut, and lungs [1].

### 2.1. Systemic Delivery

Systemic delivery introduces the drug into the bloodstream, resulting in widespread distribution of the compound primarily in vascularized tissues. There is a major advantage to using targeted systemic delivery when local injection is invasive or when the afflicted tissue is hard to reach. When diseases are not isolated to a particular area of the body, systemic delivery also holds major advantages because of the larger distribution coverage. The disadvantages of this method are that it requires relatively larger amounts of the drug or the composite vehicle/drug due to wide exposure in the plasma, blood vessels, and the vascular tissues. In addition, the sequestration in the liver and/or clearance in the kidneys may pose significant challenges for systemic drug delivery. If the drug particles are nanoscale, studies have found that less than 1% of the drug particles will reach the target tissue [2]. Thus, both drug particle sizes and blood circulation characteristics are major considerations for systemic delivery to the MSKS.

Intrinsic molecular barriers of MSK tissues for systemic delivery were studied by Ngo et al. [3]. After a bolus injection of different sized dyes into the hearts of guinea pigs, the distribution of the dye in joint tissues was analyzed. The injection consisted of high (70 kDa) and low (10 kDa) molecular weight, neutrally charged dyes, which were allowed to circulate before the knee joints were harvested and assessed for distribution. A distinct size separation was found in the tissues. Small tracers were distributed in the avascular meniscus, ligaments, and tendons but had low presence in the articular cartilage. Larger tracers were found primarily in the vascular regions such as the muscle fascia, marrow, and surrounding blood vessels. These data suggest that larger-sized particles can be delivered to vascular MSKS tissues via systemic delivery. However, systemic delivery to avascular MSKS tissues requires the infiltration of smaller-sized particles by diffusion through smaller pores in the ECM. The data also indicate that articular cartilage may be the most difficult MSKS tissue to deliver due to its avascular nature, a pore size as small as 20 nm, and the negatively charged matrix due to the high abundance of proteoglycans [4].

The direction of the blood supply may also significantly contribute to how therapeutics are delivered to target tissues in the MSKS. In a study performed by Evans et al., a directionally dependent and mechanically responsive flow from the bone to the muscle is described [2]. The periosteum subjected to a high flow rate is significantly more permeable from the bone to muscle direction rather than the muscle to bone direction. This high flow rate is mimetic of increased flow during a traumatic injury, which increases the periosteum permeability by orders of magnitude [2]. While this implication suggests that there is significant molecular and nutrient supply to support muscle health from the bone, it also may be an indication that systemically injected therapeutics will be, at least in some part, dependent on the anisotropic permeability of the periosteum. This was also validated in the previously described study by Ngo et al., where joint tissues had much higher tracer concentrations in the actual tissues and bones than the surrounding muscles [3]. The distribution of the tracers indicates the direction of the blood supply from the bone to the surrounding muscle.

In addition to blood flow and drug size, disease states may also affect systemic drug delivery. In the young, healthy guinea pigs, small channels through the articular cartilage fluoresced with the small tracer with higher overall fluorescent concentration compared with the older, arthritic animals [3]. These findings suggest not only that the size of the delivered particle alters tissue distribution but also that the disease status may affect the permeability and physiology of the affected tissue. Specifically, degenerative cartilage pathophysiology during osteoarthritis (OA) affects the small channels running through the articular cartilage into the subchondral bone, impacting OA treatment via systemic delivery [1]. Much like OA alters the delivery capacity, an injury, trauma, and disease state may increase the flow rate of the surrounding fluids, increasing the permeability of the bone [5]. Diseases such as osteoporosis and aging reduce the periosteum and bone thickness, causing the increase in permeability to nutrients and potential therapeutics [5]. With these findings, the simplest method for delivering therapeutics for treating OA would be local delivery. In OA of the knee, intra-articular injection would be the most viable method to maximize delivery to the articular cartilage. The strategies for local delivery are described as follows.

### 2.2. Local Delivery

Local drug delivery has attracted great attention for the treatment of MSKS disorders, mainly because it could deliver therapeutic agents directly to the desired site of action, provide an optimal drug level for controlled periods of time, and reduce undesirable side effects or toxicity [6]. Local delivery is often associated with increased retention of the drug depending on the target site. For example, drug particles delivered to the joint capsule can diffuse into the cartilage, meniscus, and tendons and ligaments. They will then be cleared by diffusion into the vascular synovium or through lymphatic drainage. The balance between delivery and clearance of the drug is key to maintaining half-life and efficacy.

In particular, intra-articular (IA) injection is a commonly used form of local injection to target MSKS tissues in the joint. The most common use for IA injection is for the treatment or pain management of OA. While IA injection is a promising way to reach avascular tissue of joints such as articular cartilage, disease modifying treatments are yet to be successful [7]. The rapid clearance of therapeutics injected into the joint space is a significant problem. The ideal therapeutic must have a long retention time, high local concentration, a controlled and sustainable release, and a disease-modifying or regenerative effect to compete with invasive, disease-ending procedures such as knee arthroplasty [7].

Larger particles injected into the joint are known to be phagocytized by macrophages, while smaller particles can penetrate the cartilage and the surrounding tissue; however, these smaller particles are rapidly cleared into the bloodstream. To address the need for increased retention time in the joint, a biodistribution study of nanoscale and microscale particles after intra-articular injection was conducted [7]. This study determined the fate of large- (~10 µm and ~3 µm) and small- (300 nm) sized Poly(D,L-Lactide) (PLA) particles containing a fluorescent dye. In healthy mice, both micron-sized particles had significantly higher retention time than the small, nano-sized particles. The clearance pathway for the nano-sized particles was as expected: through the blood and ultimately arriving at the liver. Once an arthritic condition was induced, the retention of ~10 μm stayed constant, while ~3 μm particles escaped from the inflamed joints, most likely due to the increased capillary permeability caused by synovial inflammation [7]. Thus, a large size is critical for drug retention within the joint space when considering an inflammatory disease state. Therein lies a dilemma where the drug particles need to be small enough to infiltrate ECM and yet large enough to retain in the joint space. Thus, considering size alone is not sufficient to create effective delivery and efficacy of a drug. Other strategies in addition to size must be considered based on specific properties of the targeted MSKS tissues.

## 3. Tissue Properties for Delivery

The MSKS tissues have varying degrees of charge density and matrix adhesive properties, which are critical factors to regulate drug delivery. In addition, drug transport through vascular routes and/or non-vascular means must be considered. Vascularity varies among MSKS tissues with high vascularity in the bone and muscle and low vascularity in the cartilage, tendon, and ligaments.

### 3.1. Vascular Tissue

Vascular tissue has blood vessel intrusions throughout, which allow nutrient, hormone, growth factor, and oxygen exchange. The two major vascular tissues of the MSKS are bone and muscle. Because of their high blood supply, targeting these tissues for drug delivery is most commonly done through systemic injection. Particles typically enter through the basement membrane and endothelial fenestra of approximately 10 μm [8]. In particular, bone receives approximately 10–15% of the total cardiac output and has the most surface area for the exchange of ions, proteins, and solutes out of any organ [8,9]. The penetration of particles into the spaces of bone after exiting the blood supply is largely based on particle size to effectively penetrate the matrix and fluid spaces. In a study by Tami et al., a variety of different-sized tracers were used to determine the penetration into the microarchitecture of bone [8]. The 0.3 kDa probes were present in the fluid space of the lacunocanalicular system and throughout the bony matrix, while the 70 kDa and larger probes were excluded from the matrix and fluid spaces entirely. For the smaller probes, mechanical loading of the tissues increased particle penetration, suggesting that loading is beneficial for targeted delivery to bone [8].

Like bone, muscle tissue is most commonly targeted via systemic delivery. Muscle tissue consists of fibers surrounded by the sarcolemma, a sheath that envelops the fibers and regulates the flow of molecules across its membrane. In order to produce efficacious results, one must overcome the limiting diffusion of the sarcolemma. In the event of an injury, small tears in the sarcolemma develop and allow transport in and out; however, these tears are quickly repaired in a healthy individual [10]. Under pathological conditions, such as muscular dystrophy, the membrane may become more fragile or have a prolonged repair time. This creates a bidirectional flow and increases the opportunities for drug molecules to reach their target within the muscle [10].

### 3.2. Avascular Tissue

Avascular tissue relies on diffusion and convection for nutrient and particle transport. These tissues in the MSKS are primarily composed of cartilage, tendons, and ligaments. The meniscus in particular can be considered to have some vascularity in the periphery after development, but the center remains avascular. Because of the lack of blood vessel perfusion, avascular tissue relies on fluid flux to transport solute to cells to maintain homeostasis and structural integrity [11,12]. Thus, avascular tissue is predominantly targeted via local delivery. While passive diffusion is the most common method of particle transport, compression-induced convection within the joint capsule can increase the rate and size of which particles can penetrate the extracellular matrix and reach the metabolically active cells [11].

Articular cartilage consists of a hydrophilic extracellular matrix composed mainly of collagen II and proteoglycans surrounding chondrocytes. The matrix carries an overall negative charge, which repels like-charged particles attempting to penetrate the narrow pores of approximately 60–200 nm in width [12,13]. Chuan Chin et al. elucidated the effect injury may have on the diffusivity of molecules into explanted cartilage tissue [12]. They used neutral dextran of 4 kDa and 40 kDa, insulin, and chondroitin sulfate to emulate protein transport. For each described solute, injury significantly increased the uptake into the tissue [12]. This was likely due to fissures produced in the matrix as well as a measured glycosaminoglycan and proteoglycan loss. This loss would reduce the negative charge of the matrix and thus the repulsive effect of negative or slightly negatively charged particles.

Meniscus tissue is similar to cartilage in that it consists of dense extracellular matrix with low cellularity. The ECM primarily consists of collagen and proteoglycans, which aid in the ability to resist compressive load. In contrast to cartilage, menisci are fibrocartilaginous. They contain fibroblast-like cells in an outer, vascular region, while the inner, avascular region contains fibrochondrocytes. After injury, there is poor cellular migration to the defect site. One way researchers have overcome this is by promoting proteoglycan or collagen degradation in the defect, which enhances cellular migration to the injury, proliferation, and new matrix synthesis.

Tendon and ligaments are viscoelastic, fibrous connective tissue. They contain very little vasculature, which requires diffusion from the synovial fluid to be the primary method of nutrient uptake [14]. Frequent mechanical stresses result in small tears on tendons and ligaments. These small tears are not easily repaired, and the defect site lacks cell migration and proliferation due to minimal vascular permeation [15]. Fibrous scarring may often occur in the injury site, resulting in altered mechanical properties and a higher likelihood of full tendon or ligament rupture [14,15]. When these ruptures occur, elastic force will pull the tendon and ligament apart from the tear region. These injuries have only been successfully repaired through primary surgery [14].

## 4. Smart Strategy for Targeting and Retention

MSKS drug delivery combined with tissue repair is attractive to researchers because when tissues suffer from a pathological variation, further damage and a longer healing time are required [16,17]. The smart delivery strategies can encompass considerations of the choices of delivery vehicle as well as the cargo to be delivered. In addition, the tissue to be targeted and the specific disease application need to be considered as well.

### 4.1. Delivery Vehicle Type Consideration

Current drug delivery vehicles being studied are largely sub-micron-sized particles classified as nanoparticles. The most common are polymer and lipid-based vehicles. They are capable of encapsulating a variety of different drug types such as nucleic acids and small molecule drugs. They are highly controllable and can encapsulate the payload via covalent interactions or electrostatic interactions. Covalent association with the payload allows for a more stable delivery system and can potentially shield the payload from enzymes, pH shifts, and other biological clearance functions. Generally, covalent bonds are cleaved upon reaching the intended site, but there are more stable alternatives if necessary. One alternative is the addition of polyethylene glycol (PEG) to shield the composite drug. This is commonly associated with intravenous injections as the composite drug requires a longer half-life to reach the target tissue [6,18,19]. Another exciting new vehicle that is being engineered for MSKS delivery is exosomes: nanovesicles derived from cells that act as system communication mediators by delivering nucleic acids and proteins to other cells [20,21]. Last, scaffolding and drug depots with polymer skeletons containing small molecule or protein drugs are gaining interest due to their ability to have a controlled, sustained release in a locally injected site [22,23,24,25].

### 4.2. Delivery Particle Size Consideration

A significant contributor to the efficacy of a drug delivery strategy is the size of the drug particles used to reach specific tissues. For example, pores of avascular cartilage such as articular cartilage have been demonstrated to vary from 60 to 200 nm. The variation of size and vascularity affects the ECM of the tissues and thus influences decisions on whether to administer the drug locally or systemically. These strategies must also account for the natural direction of blood flow and how the presence of a disease state may alter that. Several studies have been performed involving tracers of varying sizes distributed across MSKS tissues to analyze the destination and spread of the different-sized particles. Tracers in MSKS tissue around the knees showed large tracers of 70 kDa with significant presence in vascular tissues such as muscle, bone marrow, and the surrounding blood vessels [3]. This is compared with the small tracers of 10 kDa being more present in avascular tissue such as the avascular meniscus, tendons, and ligaments. Neither showed a significant presence in the articular cartilage, demonstrating a challenging region to be explored in the future. A study specifically on bone architecture found 0.3 kDa probes within the matrix and fluid of bones, while probes 70 kDa and larger were completely excluded [8]. Given the diverse nature of bone makeup, targeted drug delivery to various depths of bone tissue also leaves room to be further explored.

Analyzing the size of the particle uptake while controlling a disease or an injury state also yielded varying results. In a study on articular cartilage drug retention, particles of 10 μm and 3 μm were injected, and the induction of an arthritic state caused an increased 3 μm loss, while the 10 μm particles were retained [7]. The results in a crush injury model demonstrated different properties from the previous arthritic models. A study modeling crush injury using cartilage explants saw increased uptake of 4 kDa tracers, 40 kDa tracers, and other associated proteins and molecules used in the experiment [12]. This concept was reinforced with the findings of another study that credits compressive-induced convection with allowing a greater size of particles a greater rate of induction within a joint capsule [11]. The combination of these results could show the large role of compressive forces in particle diffusion and convection, especially in cartilage. When compared with studies on drug retention and arthritic disease states, these results could cause research on MSKS drug delivery to fork in different directions depending on the nature of the disease state.

### 4.3. Delivery Particle Surface Charge Consideration

Typically, the ECM of MSKS tissues such as cartilage holds a negative charge, making positive-charged drug delivery vehicles preferrable [12,13]. The delivery goal should be to enter the tissue and stay for sustained drug delivery. Too small of a nanoparticle will be drained, while too large or negatively charged will not traverse the pores of the ECM. A promising direction of MSKS drug delivery is demonstrated by Xu et al. [26]. Their research embedded drug particles in a hyaluronic acid (HA)-PEG shell combined with hydroxyapatite to form a drug delivery nanoparticle. This combination of the organic component of HA and the inorganic hydroxyapatite and PEG demonstrated sustained drug release combined with the ability to target osteosarcomas. The sizes of the nanoparticles used were 182.5 nm at their largest, which matches the distribution of smaller tracers being more present in the lacunocanalicular system and demonstrating potential applications of hybrid particles in avascular tissues as well [8]. Xu et al.’s nanoparticle mesh has a promising strategy of converting the barrier of delivery to the carrier to create carrier interactions [26]. The barrier to carrier strategy will increase interactions between the drug delivery vehicle and the target site tissue, increasing drug delivery particle retention and thus improving delivery. Depending on the charge and structure of the drug or composite, adjustments can also be made with the hybrid components of the nanoparticle to better match the target barrier.

A large hurdle to overcome with drug delivery systems is the adsorption of biomolecules. In the case of nanoparticles, protein coronas can result in altered surface properties, thus hindering the targeting capacity of said particle [25]. Short-term incubation in serum results in the adsorption of enough proteins to a nanoparticle’s surface that the overall charge switches from cationic to anionic [25]. This association also reduces the nanoparticle stability, resulting in aggregates and reduced matrix penetration due to an increase in the hydrodiameter and the neutralization or conversion of charge. Although a cationic charge is important for matrix penetration and the cellular uptake of cargo, it is also known that positively charged nanoparticles exhibit lower cytocompatibility compared with neutrally or negatively charged particles [25]. Thus, it becomes extremely important to understand how the delivery vehicle will interact with the biomolecules it may be exposed to in its particular route of delivery. The protein composition remains similar when comparing the joint capsule with the serum. However, the protein concentrations and the time before reaching the target both vary between the two settings.

### 4.4. Delivery Particle Internal Property Considerations

It is often preferable to have a slow and sustained release of drugs, which could be achieved by the modification of the internal properties of delivery vehicles. A layer by layer fabrication of an elastomer with a drug encapsulated was developed by Wei et al. [27]. This prototype demonstrated potent wound healing capabilities under cyclic deformation, creating potential applications for use for injuries in mechanically active areas. A lipid nanoparticle-based hydrogel loaded with curcumin is reported by Jiang et al. to reduce the chronic musculoskeletal pain via a local delivery method [28]. The targeted delivery to the MSKS demonstrates the innovation of methods of drug delivery specific to MSKS tissue. More recently, J. Park et al. demonstrated an oral delivery of growth factor that targeted in MSKS cell proliferation, via the encapsulation of plant cells [29]. This approach overcomes the hinderance of traditional delivery of growth factors that acquire injections or local implantation, while demonstrating methods for targeting the MSKS through the GI tract [29]. Braun et al. developed a matrix metalloproteinase (MMP) responsive delivery system specific to skeletal muscle cells that releases myostatin inhibitors in response to MMP activity in muscle [30]. This bioresponsive drug release demonstrates modification of the internal properties of the delivery vehicle and optimization of treatment while maintaining tissue-specific targeting ability.

### 4.5. Delivery Cargo Considerations

The drug target specificity can be achieved by biologics including peptide and nucleic acid. When discussing deliverable cargo, proteins can target receptors and binding partners, while nucleic acids such as small interfering RNA (siRNA) or microRNA are of great interest due to their ability to selectively regulate gene targets. These gene targets may be highly expressed in disease tissue and can be knocked down by an effective delivery of siRNA or microRNA [18,31]. However, delivery vehicles are required for efficient cellular uptake because of the overall negative charge of the nucleic acids as well as their instability when exposed to biomolecular or immune interactions. Small molecule drugs are another common cargo with MSKS delivery vehicles. The extremely small size (<10 nm) allows unspecified delivery and the potential for off-target effects. These drugs are often conjugated with targeting moieties or encapsulated within vehicles to improve specificity [23,30,32]. Last, growth factors and other protein-based drugs are encapsulated or conjugated to vehicles for the same stability and specificity purposes [20,33,34]. The following categories seek to elucidate methods to enhance delivery to the described MSKS tissue.

## 5. Tissue-Specific Considerations

### 5.1. Bone

Bone is the most widely studied MSKS tissue for targeted delivery. The unique mineral content and various cell types offer opportunities for many targeting strategies. Because bone delivery is primarily through systemic administration, it is essential to increase the bioavailability of the drug to the target tissue. The most commonly used ligands are bisphosphonates due to their high binding affinity to hydroxyapatite [35]. These drugs are especially useful in treating post-menopausal osteoporosis as a replacement for hormonal replacement therapy (HRT) in patients who either refuse or are not suitable for it [36,37]. However, long-term use of bisphosphonates may result in negative side effects in a variety of organ systems [38]. Thus, targeting specific cell types or proteins has become of higher interest, especially in the case of osteoporosis. One example is denosumab, which targets the interaction of RANK to RANKL, disrupting bone resorption and treating osteoporosis in the setting of glucocorticoid induction as well as post-menopause [39,40]. Although more data should be collected, the drug has performed well in phase II and III clinical trials with decreased side effects and higher preference compared with current bisphosphonates [39,40,41]. The most promising drug and furthest along is romosozumab, a monoclonal antibody that binds to and inhibits sclerostin [42]. Sclerostin is a key inhibitor of bone formation, and its inhibition will still allow for the body to restore skeletal architecture rather than simply blocking bone resorption as seen in many current strategies [43]. However, strategies for inhibiting bone resorption are also showing promising results to increase bone fracture healing. Two separate studies by Newman et al. and Wang et al. both use TRAP targeting polymers to increase the bone affinity of an injection or to deliver a GSK3b inhibitor, respectively [1,44,45].

### 5.2. Muscle

While bone drug delivery focuses on targeting motifs, muscle targeting delivery vehicles are mainly focused on conjugating ligands to bind surface recognition elements. Carnitine conjugates have been shown to increase muscle uptake [46]. However, carnitine has also been shown to have specificity to the kidney [47]. To target muscle tissue more specifically, Braun et al. developed a MMP cleavable linker to release myostatin inhibitor for the treatment of sarcopenia [30]. In the event of a flare-up, MMP-9 will be significantly upregulated in the muscle. Once the drug reaches the inflamed muscle tissue, MMP-9 will cleave the protease cleavable linker (PCL) and release myostatin inhibitor to drive the regeneration of functional skeletal muscle tissue [30]. Some of the drugs under development for targeting bone and muscle diseases are listed in Table 1.

### 5.3. Cartilage

Cartilage is considered as a premier example for drug delivery to avascular tissues. The delivery method is almost universally local administration. Most commonly, drugs are delivered into the joint capsule by intra-articular injection. With osteoarthritis being the most common cartilage ailment, there is a clinical need to fill the gap of disease modifying drugs. The problem that researchers face likely stems from the lack of simultaneous retention of both small molecule and macroparticle drugs due to the clearance from the joint cavity via synovial capillaries and lymph drainage [13]. This retention time is combated in two ways. The first is using either large delivery vehicles such as scaffolds or drug depots. The second is to explore nanoparticles with aggregating properties that prevent them from being flushed out of the joint space. However, large particles have difficulty penetrating the cartilage ECM, which has a small pore size. Binding and/or aggregation of biomolecules due to a prolonged retention may cause alteration of the surface properties of ECM such as charge. A smart strategy is to have a delivery vehicle small enough to penetrate the ECM and then be retained within the ECM for the sustained release of the cargo.

### 5.4. Meniscus

While the meniscus does have a vascular region, the vascular permeation decreases to a very minimal amount in adults and even more through aging. Because of this, the primary drug delivery strategy is local administration. Biomaterials are the predominantly studied therapeutic candidate consisting of bioadhesives, cell migration promoters, and proliferation promoting scaffolds. Qu et al. adopted an interesting approach by developing an electrospun polyethylene oxide scaffold containing collagenase, a collagen-targeting degradative enzyme [64]. When hydrated in the articular space, the scaffold would swell and release collagenase to reduce the density of the ECM, which resulted in an increase in cellular migration and wound regeneration [64]. This innovative approach utilized the injury targeting capabilities of collagenases to stimulate native cells to repair the damaged tissue.

### 5.5. Tendon and Ligament

There is limited research and an unmet clinical need of targeted drug delivery to repair damaged tendons or ligaments. The poor vascularity of these tissues likely points to local administration being the most effective delivery method [15]. There is no targeting method being studied currently, and most treatment options rely on the general administration of anti-inflammatory pharmaceuticals [65]. However, collagen and fibrin gels for local injection have been discussed as potential drug depots for controlled release [15]. There have also been past studies using non-targeting microbubbles to encapsulate and deliver genes to tendons in mice. Delalande et al. used these microbubbles to deliver fibromodulin plasmid DNA to the Achilles tendon of fibromodulin knockout mice. This resulted in the regeneration of the degenerative tendon [66].

## 6. Summary and Future Direction

The musculoskeletal system is composed of a wide variety of tissues designed to meet the human body’s range of motor and structural needs. This variety, while helpful in everyday life, presents a unique challenge for drug delivery and the treatment of injuries. The varying size and vascularity of MSKS tissues are dictated by their wide variety of structure and function. This variation necessitates different goals for targeting motifs. Avascular tissues and local delivery mainly require prolonged retention time; however, the penetration of the matrix is necessary as well, and achieving penetration and retention simultaneously is difficult. Vascular tissues would greatly benefit from targeting motifs, especially in systemic delivery. Cellular or enzyme responsive targeting would be ideal given the nature of the surrounding extracellular matrix coupled with blood flow.

Considerations for drug delivery into the MSKS include the following: (1) particle size plays a crucial role in both systemic and local delivery to penetrate the matrix effectively. (2) Compressive forces play a large role, especially for cartilage penetration, in particle diffusion and convection. (3) Targeting motifs are especially important in systemic delivery to vascular tissue. They can benefit greatly from cellular or enzyme responsive targeting. (4) Avascular tissues and local delivery mainly require prolonged retention time; however, the penetration of the matrix is necessary as well.

Tissues of interest can also be better targeted by adding targeting moieties to the drug or to a delivery vehicle encapsulating the drug. There is much room for exploration given the variety of tissues and tissue structures, necessitating a vast selection of modifications to match the charge, size, disease state, and delivery location. Studies in the current literature have demonstrated appropriate nanoparticle sizes, compositions, and administration, providing parameters to strive toward in future research. They have also laid the groundwork on exploring new types of nanoparticles with promising potential. The diverse properties of the musculoskeletal system provide a unique challenge to future studies improving drug delivery. In addition, the disease pathophysiology will greatly influence the therapeutic delivery and must be taken into account when assessing the route of administration and therapeutic composition. Because of these differences, the route of administration will be crucial to reach the tissue of interest. The tissue of interest can be better targeted by adding targeting moieties to the drug or to a delivery vehicle encapsulating the drug. The drug vehicle and cargo must be appropriately sized and charged to navigate the matrix in that specific tissue. Thus, smart strategies for targeted drug delivery are required for treating MSKS-specific diseases.

## Figures and Tables

**Table 1:** Examples of systemic delivery of drugs targeting bone and muscle.

Tissue	Compound	Category	Target	Application
Bone	Alendronic acid	Bisphosphonate	Hydroxyapatite	Osteoporosis (BMD maintenance) [4,36,37]
	Zoledronic acid	Bisphosphonate	Hydroxyapatite	Hypercalcemia [35,48] Osteoporosis [49,50,51]
	PEG-zoledronic acid-PLGA	Bisphosphonate/NP conjugate	Hydroxyapatite	General bone targeting [26,52]
	PEG-aspartic acid-liposome	Oligopeptide/NP conjugate	Ca2+	Bone metastasis [53]
	Romosozumab	Monoclonal antibody	Sclerostin	Osteoporosis [42]
	TBP-NP	Peptide targeting linker	TRAP	Fracture healing [45]
	Denosumab	Monoclonal antibody	RANKL	Osteoporosis [39,40,41] Rheumatoid arthritis [54,55]
Muscle	Carinitine	Transporter/therapeutic conjugate	OCTN2	General muscle targeting [56,57]
	PCL-myostatin	Protease cleavable linker/therapeutic conjugate	(MMP) 1, 8, 9 activities	Myositis [30]
	OX26mAB-immunoliposome	Monoclonal antibody/NP conjugate	Tranferrin receptor	General muscle targeting [58]
	M12-PMO	Muscle specific peptide/Morpholino conjugate	Skeletal muscle *	DMD [59]
	3E10mAB	Monoclonal antibody/therapeutic conjugate	Double-strand DNA; ENT2 transport	X-linked myotubular myopathy [60] Pompe disease [61]
	ASSLNIA	Muscle-specific peptide/plasmid conjugate	Skeletal muscle *	General muscle targeting [62]
	A01B	RNA aptamer/NA	Aptamer/therapeutic conjugate	DMD [63]

* Specific target beyond scope of study. PEG: poly (ethylene glycol); PLGA: poly(lactic-co-glycolic acid); TBP: TRAP binding protein; NP: nanoparticle; NA: nucleic acid; PCL: protease cleavable linker; PMO: phosphorodiamidate morpholino; BMD: bone mineral density; DMD: Duchenne muscular dystrophy.

## Data Availability

Not applicable.
